# Interferon-induced sarcoidosis with uveitis as the initial symptom: a case report and review of the literature

**DOI:** 10.1186/s13256-021-03181-x

**Published:** 2021-11-27

**Authors:** Ai Kato, Mami Ishihara, Nobuhisa Mizuki

**Affiliations:** Department of Ophthalmology and Visual Science, Yokohama City University School of Medicine, 3-9 Fukuura, Kanazawa-ku, Yokohama, Kanagawa 236-0004 Japan

**Keywords:** Interferon, Sarcoidosis, Uveitis, Interferon-induced sarcoidosis, Case report

## Abstract

**Background:**

In recent years, numerous studies have reported the development or exacerbation of sarcoidosis due to interferon therapy. However, ocular lesions rarely present as initial symptoms. Herein, we describe a rare case of interferon-α-induced sarcoidosis with uveitis as the initial symptom, and present a review of the relevant literature.

**Case presentation:**

This case involved a 62-year-old-Japanese woman with a history of a combination treatment of pegylated interferon-α-2a, ribavirin, and simeprevir, after which she developed granulomatous panuveitis. She was subsequently diagnosed with sarcoidosis following histological examination of skin biopsy specimens. In addition to reporting this case, we performed a literature review of 27 cases (24 case reports) of histopathologically diagnosed interferon-α-induced sarcoidosis published between January 2009 and November 2018.

**Conclusions:**

Among the reviewed cases, 23 (85.1%) cases developed skin lesions and 19 (70.1%) had lung lesions. Only three cases (11.1%) had accompanying eye lesions. Interferon-α therapy was discontinued in 16 cases (52.9%), and the majority exhibited improvement after systemic corticosteroid treatment. There are few reported cases of interferon-α-induced sarcoidosis with uveitis as the initial symptom. However, if uveitis develops during or after interferon-α treatment, it might represent an initial symptom of interferon-α-induced sarcoidosis, as observed in the present case.

## Background

Interferon (IFN) is an important therapeutic agent for treating chronic hepatitis C. There have been an increasing number of reports on the development or exacerbation of sarcoidosis lesions in recent years due to IFN therapy. It is well established that cell-mediated immunity by T-helper type-1 (Th1) cells is involved in the development of sarcoidosis [1, 2]. However, a Th1/T-helper type-2 (Th2) imbalance due to IFN administration and a shift to Th1 may also result in disease development, although the mechanism is unknown [3]. Skin and pulmonary lesions are the most commonly manifested initial lesions of IFN-α-induced sarcoidosis [4], whereas ocular lesions rarely present as initial symptoms [5].

Herein, we describe a case of IFN-α-induced sarcoidosis with uveitis as the initial symptom, and present a review of IFN-α and IFN-β-related case reports published between January 2009 and August 2018.

## Case presentation

The patient described in this report provided written consent for the publication of her data.

**Fig. 1:**
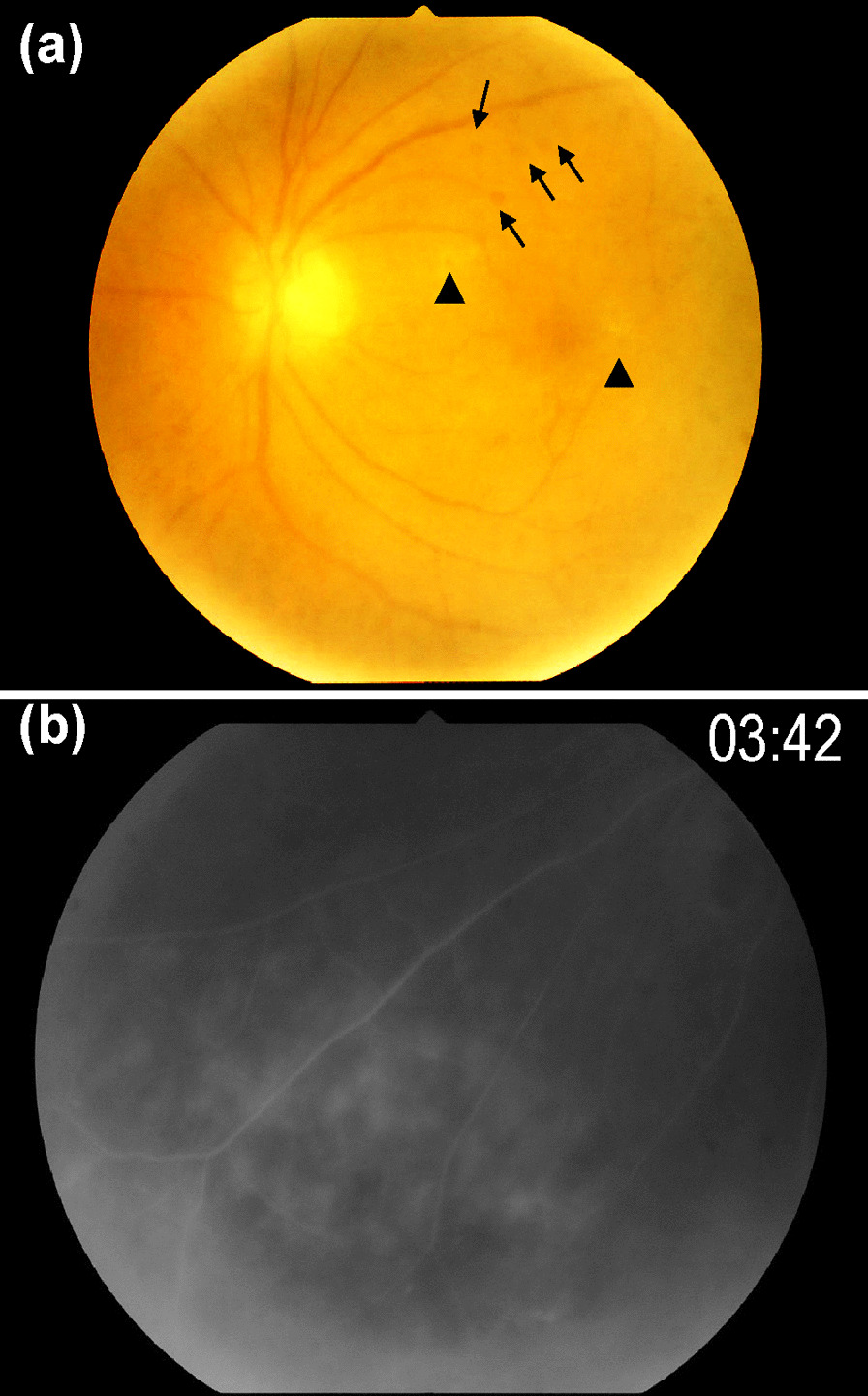
Imaging findings of a 62-year-old woman with interferon-induced sarcoidosis and uveitis. **a** Fundus photograph of the left eye shows vitreous opacity, chorioretinal exudates (arrowheads), and retinal hemorrhage (arrows). **b** Fluorescein angiography of the left eye shows fluorescein leakage from the retinal vein and hyperfluorescence consistent with the exudates

A 62-year-old-Japanese woman presented with a 20-year history of hepatitis C. She did not report any significant family or psychosocial history. From October 2014 to March 2015, she was on the following three-drug combination regimen: pegylated IFN-α-2a (180 μg/week for 24 weeks), ribavirin, and simeprevir. Subsequently, combination therapy with sofosbuvir and ledipasvir was administered from July 2016 to September 2016. In November 2016, she presented with blurred vision in the right eye and was diagnosed with anterior uveitis. Her condition improved after the topical administration of corticosteroids at a nearby eye clinic. In January 2017, she developed panuveitis in the left eye. She was treated with topical corticosteroids but without any relief. Hence, she was referred to our hospital for the treatment of blurred vision. At her first visit, her best-corrected visual acuity was 20/20 in both eyes and her intraocular pressure was 16/15 mmHg. There was no inflammation in the anterior chamber, although a tent-like peripheral anterior synechia was observed in both eyes. Chorioretinal exudates and optic disc hyperemia were observed in the right eye, whereas vitreous opacity, retinal periphlebitis, chorioretinal exudates, and retinal hemorrhages were observed in the left eye (Fig. 1a) Fluorescein angiography revealed significant fluorescein leakage from the retinal vein and hyperfluorescence consistent with the exudates in the periphery of the left fundus (Fig. 1b) No macular edema was observed on optical coherence tomography.

**Fig. 2:**
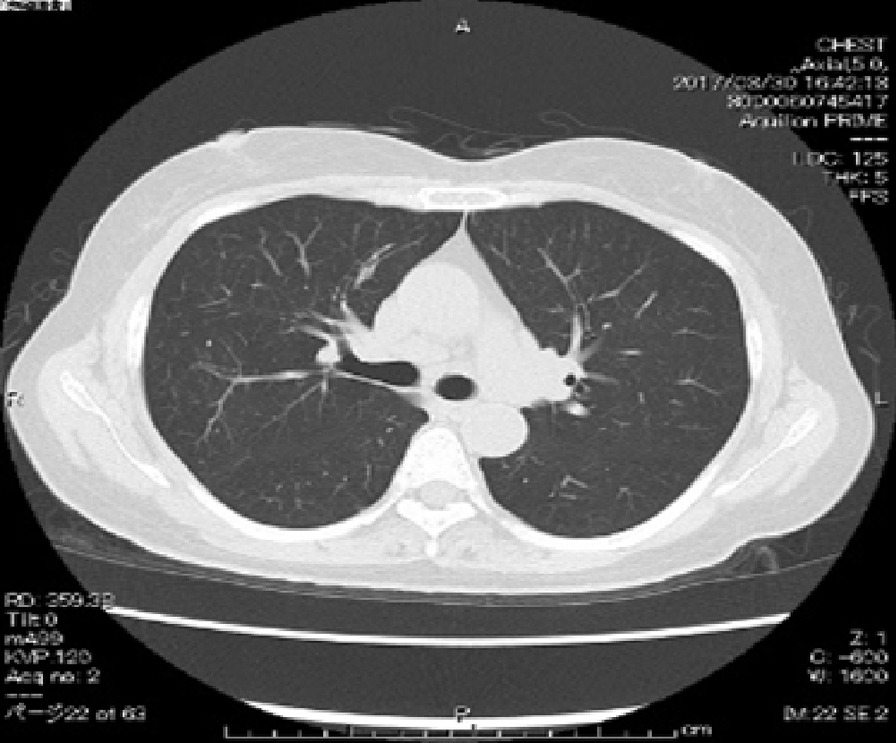
Chest computed tomography. Hilar and mediastinal lymph node swelling and numerous nodular lesions are seen in the bilateral middle lung lobes

The patient’s blood count and angiotensin-converting enzyme level were normal [20.6 U/mL (normal range 7.0–25.0 U/mL)]; however, her soluble interleukin (IL)-2 receptor level was high [1214 U/mL (normal range 145–519 U/mL)]. The patient tested positive for hepatitis C virus antibody, whereas she was negative for anti-human T-cell leukemia virus type 1 antibody; she also had a negative syphilis test and IFN-γ release assay (for tuberculosis). On chest computed tomography, swelling of the hilar and mediastinal lymph nodes, and numerous nodular lesions in the bilateral middle lung lobes and from the middle lung regions to the pleural surface were detected (Fig. 2). Gallium-67 scintigraphy showed increased radiotracer accumulation in the bilateral hilar region and mediastinum, as well as light accumulation in both thighs.

**Fig. 3:**
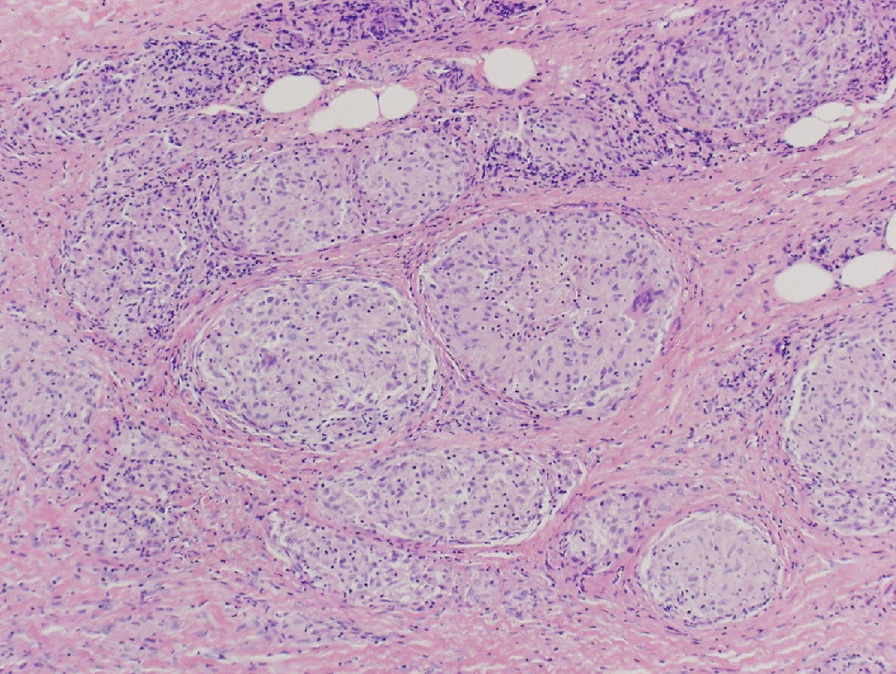
Histological examination of the skin biopsy specimen reveals noncaseating granulomas (hematoxylin-eosin × 100).

Examination of the bronchoalveolar lavage fluid showed an increased lymphocyte ratio, with a CD4^+^/CD8^+^ ratio of 5.27. No noncaseating granuloma were observed on transbronchial lung biopsy. At the beginning of February 2017, a skin eruption appeared in the right femoral region, which increased in size progressively. Histological examination of a skin biopsy sample revealed noncaseating epithelioid cell granulomas consistent with sarcoidosis (Fig. 3). The vitreous opacity in the left eye deteriorated despite betamethasone phosphate instillation. In May 2017, the patient received sub-Tenon’s triamcinolone acetonide injection in the left eye, which improved the vitreous opacity. Cataract surgery for each eye was performed in April and September 2018, and the inflammation had not exacerbated when this report was written. The pulmonologists in our hospital continued her follow-up without treatment, considering that the lung lesions did not reveal any significant change. The skin lesion disappeared within a few months without any treatment.

## Discussion and conclusions

We reviewed 24 case reports (27 cases) of IFN-α-induced sarcoidosis (Table 1) [5–28] and 6 case reports (6 cases) of IFN-β-induced sarcoidosis (Table 2) [29–34] that were published between January 2009 and November 2018. All the cases were diagnosed based on histological evaluation.

**Table 1.** Twenty-seven reported cases of IFN-α-induced sarcoidosis [5–28].

		Patients (*n*)	Proportion of patients (%)
Primary diseases	Chronic hepatitis C	24	88.9
	Melanoma	2	7.4
	Erythrocytosis	1	3.7
Sex	Male	12	44.4
	Female	15	55.6
Age of sarcoidosis onset (mean ± standard deviation)	Overall: 51.4 ± 10.8 years Men: 48.3 ± 9.9 years Women: 53.9 ± 11.1 years	–	–
Treatment	IFN-α + ribavirin	21	77.8
	IFN-α	4	14.8
	IFN-α + ribavirin + others	2	7.4
Period until onset	7.9 months (*n* = 24) (range, 2–14 months)	–	–
Organ involvement	Skin	23	85.1
	Lung*	19	70.1
	Lymphadenopathy (mediastinal)	11	40.7
	Lymphadenopathy (hilar)	11	40.7
	Lymphadenopathy (axillary)	11	40.7
	Eye	4	14.8
	Other (heart, liver, central nervous system, retroperitoneal lymphadenopathy, stomach, thyroid)	3 Each 1	11.1 Each 3.7
Therapy	Discontinuation of IFN	16	59.2
	Continuation of IFN	2	25.9
	Already completed IFN treatment	2	7.4
	Unknown	2	7.4
	Topical steroid	10	37.0
	Oral steroid	8	29.6
	Steroid pulse	2	7.4
	Others (permanent cardiac pacemaker)	1	3.7
Outcome	Resolution	15	55.6
	Improvement	8	29.6
	No change	2	7.4
	Death	1	3.7
	Unknown	1	3.7

*IFN* interferon, *n* number

^*^ with or without mediastinal and/or hilar lymphadenopathy

**Table 2.** Six reported cases of IFN-β-induced sarcoidosis [29–34].

		Patients (*n*)	Proportion of patients (%)
Primary diseases	Multiple sclerosis	5	88.3
	Melanoma	1	16.7
Sex	Male	6	33.3
	Female	2	66.7
Age of sarcoidosis onset (mean ± standard deviation)	Overall: 40.2 ± 11.1 years Men: 48.0 ± 18.4 years Women: 36.3 ± 6.8 years	–	–
Period until onset	53.8 months (range, 5–156 months)	–	–
Organ involvement	Lung*	6	100
	Lymphadenopathy Hilar	5	83.3
	Mediastinal	4	66.6
	Abdominal	1	16.6
	Skin, spleen	Each 2	Each 33.3
	Liver	1	16.6
Therapy	Discontinuation of IFN	6	100
	Oral steroid	5	83.3
	Other medicine	3	50
Outcome	Resolution	4	66.7
	Improvement	1	16.6
	No change	1	16.6

*IFN* interferon, *n* number

^*^With or without mediastinal and/or hilar lymphadenopathy

The primary diseases of IFN-α-induced sarcoidosis were chronic hepatitis C in 24 cases, melanoma in 2, and erythrocytosis in 1 case. The male-to-female ratio was 4:5. The mean age at sarcoidosis onset was 51.4 ± 10.8 years (men 48.3 ± 9.9 years; women 53.9 ± 11.1 years). Twenty-three cases (85.1%) developed skin lesions and 19 (70.1%) had lung lesions. Only three cases (11.1%) presented with accompanying eye lesions [5–7]. After initiation of IFN-α therapy, the average duration of sarcoidosis onset was 7.9 months (range 2–14 months; Table 1). IFN-α therapy was discontinued in 16 patients, and most of them showed improvement after systemic corticosteroid treatment; however, one patient died because of central nervous system disease (Table 1) [8]. Six cases of IFN-β-induced sarcoidosis are presented in Table 2. The primary diseases of these IFN-β-induced sarcoidosis cases were multiple sclerosis in five cases and melanoma in one case. The lung was the most common site of disease development in six cases (100%), and none of the cases presented with eye lesions. In addition, after IFN-β therapy, the average duration of sarcoidosis onset was 53.8 months (range 5–156 months). The duration of sarcoidosis onset was longer in the IFN-β therapy group than in the IFN-α therapy group (*p* = 0.094; *t*-test).

Our patient developed uveitis 25 months after initiating combination therapy with IFN-α and ribavirin to treat chronic hepatitis C. After 5 months, she was histologically diagnosed with sarcoidosis following a skin biopsy of the eruption on the right thigh. Uveitis was controlled with topical corticosteroid treatment.

Sarcoidosis is a multisystemic granulomatous disease. In Japan, the frequency of pulmonary, eye, skin, or heart lesions is high [35]. Th1 cell-mediated immunity is thought to be involved in the onset of the disease. IFN-α works in combination with IL-12, involving a mechanism wherein IFN-α administration causes increased production of IFN-γ and IL-18 [2, 3]. This may shift the immune balance to Th1 dominance.

In contrast, ribavirin may suppress the Th2 immune response and activate the Th1 immune response. Although ribavirin-induced sarcoidosis has not been reported, combination therapy with IFN-α and ribavirin could increase sarcoidosis susceptibility. In addition, sarcoidosis has been reported to develop in patients with chronic hepatitis C without IFN-α treatment [36]. Therefore, the hepatitis C virus itself may be a trigger for the onset of symptoms. From the literature, we learned that IFN-α was associated with a shorter time of sarcoidosis onset than IFN-β (7.9 versus 53.8 months). Both hepatitis C virus infection and IFN-α therapy may shorten sarcoidosis onset compared with IFN-β. It is unclear if our patient developed sarcoidosis after a longer course (25 months) than the mean onset period of IFN-α-induced prolapse.

While the most common symptom of IFN-α-induced sarcoidosis was skin lesion (Table 1), there have been few cases with uveitis in the reported literature [5–7]; three of four cases, including our case, involved Japanese patients. Uveitis appears more common among Japanese patients with IFN-induced sarcoidosis owing to the greater frequency of eye involvement in sarcoidosis among Japanese patients [35] than non-Japanese patients [37]. Uveitis developed as an initial symptom in the present case, while skin lesions with uveitis appeared together in the case reported by Nigam *et al*. [5]. In other Japanese cases [6, 7], skin lesions developed before uveitis.

Few reports have outlined the treatment of IFN-induced sarcoid uveitis. One reported case of IFN-α-induced sarcoid uveitis with papillitis and macular edema showed improvement following sub-Tenon’s injection of methylprednisolone [5]. Doycheva *et al*. [38] reported treatment for a mild case of IFN-α-induced sarcoid panuveitis in which, despite the absence of histological examination, the dosage of IFN-α was gradually reduced and discontinued and replaced with steroid eye drops. In contrast, in cases with systemic symptoms, IFN-α was discontinued and oral steroids were needed [38]. In our case, the patient’s condition improved following local steroid treatment alone, including steroid eye drops and sub-Tenon’s triamcinolone acetonide injection, although the lung lesion persisted.

Our literature review has some limitations. First, we only verified articles that could be searched and read; thus, the actual number of articles may be greater. Second, we only included cases of histopathologically confirmed IFN-induced sarcoidosis and did not consider clinically diagnosed cases. Thus, the number of cases might have been underestimated.

When using IFN treatment, it is necessary to monitor the onset of symptoms of sarcoidosis and systemic side effects of IFNs. If uveitis develops during or after IFN treatment, it may be the initial symptom of IFN-induced sarcoidosis. Ophthalmologists should suspect the development of sarcoidosis and conduct systemic investigations to diagnose sarcoidosis.

## Data Availability

The dataset supporting the conclusions of this article is included within the article.

## Abbreviations

IFN: Interferon
IL: Interleukin
Th1: T-helper type-1
